# High connectivity and low differentiation of *Plasmodium falciparum* parasite populations in a setting with high seasonal migration

**DOI:** 10.1038/s41598-025-29271-0

**Published:** 2025-11-26

**Authors:** Endashaw Esayas, William Louie, Isobel Routledge, Maxwell Murphy, Nigatu Negash Demeke, Faith De Amaral, Andrés Aranda-Díaz, Bryan Greenhouse, Fikregabrail Aberra Kassa, Tedros Nigusse, Muluken Assefa, Temesgen Ashine, Asefaw Getachew, Henry Ntuku, Lemu Golassa, Endalamaw Gadisa, Adam Bennett, Jennifer L. Smith

**Affiliations:** 1 Malaria and Neglected Tropical Diseases Research Division, Armauer Hansen Research Institute, Addis Ababa, Ethiopia; 2 Aklilu Lemma Institute of Pathobiology, Addis Ababa University, Addis Ababa, Ethiopia; 3 EPPIcenter Research Program, Division of HIV, Infectious Diseases and Global Medicine, Department of Medicine, University of California, San Francisco, San Francisco, CA USA; 4 Malaria Elimination Initiative, Global Health Group, University of California San Francisco, San Francisco, CA USA; 5 PATH Malaria Control and Elimination Partnership in Africa (MACEPA), Seattle, USA

**Keywords:** Malaria, *Plasmodium falciparum*, Amplicon sequencing, Ethiopia, Highland-lowland populations, Seasonal agricultural workers, Genetic connectivity, Complexity of infection, Biological techniques, Computational biology and bioinformatics, Diseases

## Abstract

Seasonal movement of less-immune people from low- to high- transmission regions increase malaria risk and may introduce parasite strains to both areas. This study examined *Plasmodium falciparum* genetic diversity and connectivity between low-transmission highlands and endemic lowlands in Ethiopia to assess the contribution of seasonal agricultural migration in sustaining transmission. *P. falciparum* qPCR-positive dried blood spots collected from highland health facilities and lowland agricultural worksites were sequenced using multiplexed amplicon sequencing. Complexity of infection (COI) and infection pairwise relatedness were estimated and used for clustering analysis. Lowland populations (seasonal workers and residents) had higher COI and polyclonal infection rates (mean COI 2.62, 62%, n = 581) than highland residents (mean COI 2.00, 40%, n = 599). Similar expected heterozygosity (He ≈ 0.4) was observed, and *P. falciparum* infections from worksites showed high genetic connectivity between highland and lowland populations, with extensive parasite sharing, including 27 related clusters in highland cases and 10 in seasonal workers. Integrating parasite genomic data with epidemiological information revealed strong connectivity and low genetic differentiation between these regions linked by seasonal migration. These findings highlight how agricultural mobility likely drives parasite diversity and gene flow, implicating its role in sustaining malaria transmission.

**Supplementary Information:**

The online version contains supplementary material available at 10.1038/s41598-025-29271-0.

## Introduction

Malaria remains a significant public health challenge in Ethiopia, with complex transmission dynamics influenced by diverse eco-topographies, vector populations, local weather patterns, and human movement^1,2^. Roughly 70% of the population live in areas of transmission^3^, which is generally unstable and heterogeneous across different altitudinal zones^4^, with outbreaks occasionally leading to widespread transmission increases. Population movement—primarily driven by seasonal migrant workers seeking agricultural employment—is a major factor sustaining malaria transmission in low-endemic regions of Ethiopia^5,6^. Each year, over 500,000 individuals migrate from the highlands to high-transmission lowland agricultural areas within the Amhara region during the farming season^4^. This creates seasonal hubs where both migrant workers and residents are exposed to malaria and may contribute to the connectivity of parasite populations between regions^5,7,8^.

Seasonal agricultural workers in northwestern Ethiopia often face increased vulnerability to malaria due to heightened occupational exposure to infectious mosquito bites and suboptimal housing conditions commonly found at agricultural worksites^8–11^. Seasonal migration not only elevates individual risk but also facilitates the movement of malaria parasites from higher endemic lowlands to highland communities, potentially sustaining parasite reservoirs and triggering localized outbreaks^12,13^. Populations in these highland areas, with less prior exposure to malaria, lack protective immunity, increasing the risk of severe malaria, especially among children and pregnant women^6,14,15^. Further compounding this vulnerability, recent studies have identified both primary and secondary malaria vectors in the highland^16,17^, raising concerns about the potential for rapid transmission resurgence—patterns observed during the period of this study. Therefore, understanding that parasite migration mirrors human migration is crucial for devising more effective malaria control strategies in regions with high mobility and complex transmission dynamics, including the identification of high-risk areas for parasite introduction^12,13,18^.

Travel to higher endemic regions is a common risk factor for malaria, but it remains an indirect and imprecise measure of parasite spread, subject to various biases^6,19,20^. Recent advancements in next-generation sequencing and methods targeting highly diverse loci within the *P. falciparum* genome, coupled with novel analytical techniques, now allow for the measurement of fine-scale parasite genetic diversity and population structure^21^. When integrated with epidemiological data, these genomic insights can quantify parasite relatedness and population differentiation^22,23^, offering new opportunities for more effective malaria control and elimination strategies. This is especially important in regions with complex transmission dynamics, such as Ethiopia, where seasonal migration plays a significant role in parasite spread^12,13^. Despite these advances, to date, few studies have leveraged genomic tools to explore parasite connectivity between highland and lowland areas.

This study aimed to evaluate the impact of seasonal agricultural worker movement on transmission dynamics by examining the genetic diversity, population structure, and connectivity of *P. falciparum* populations between Ethiopia’s low-transmission highland and high transmission lowland areas. Genomic epidemiology provides a valuable layer of quantitative data, revealing transmission patterns that traditional methods may miss. By inferring parasite flow and importation rates, these insights can guide the development of targeted malaria control strategies and inform decision-making, particularly in regions with high population mobility.

## Methods

### Study site and population

**Fig. 1:**
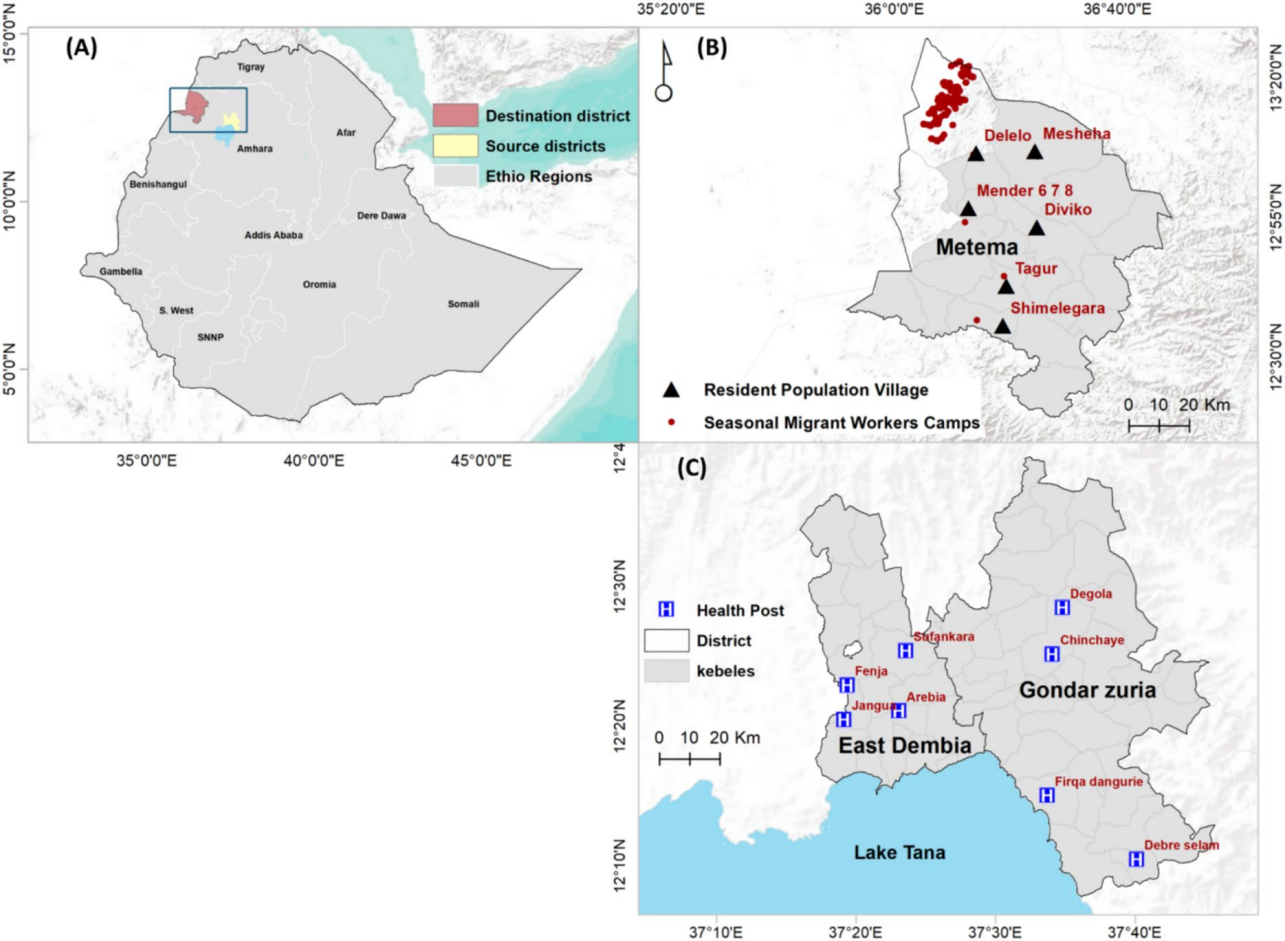
Map of the study area in northwestern Ethiopia. (**a**) Administrative map of Ethiopia highlighting source and destination districts of seasonal migrant workers. Ethio Regions: Ethiopian region. (**b**) Lowland areas showing seasonal migrant workers camps and resident population villages. (**c**) Health posts selected from Highland resident areas. Map was created using QGIS version 3.44.2 “Solothurn” (QGIS Development Team, 2025; https://qgis.org). (GIS Development Team (2025). *QGIS Geographic Information System*, version 3.44.2 “Solothurn”. Open-Source Geospatial Foundation Project. Available from: https://qgis.org).

The study was conducted in three districts of northwestern Ethiopia: two highland districts (Gondar Zuria and East Dembia) and one lowland district (Metema) (Fig. 1). Formative and human behavioral observation studies^8^, along with previous research, guided the selection of study sites, which are characterized by large numbers of seasonal migrant workers in the highlands and historically high malaria incidence in the lowlands^24,25^. Gondar Zuria and East Dembia are the highland residential areas where many migrant workers reside permanently. These workers travel seasonally to lowland agricultural destinations, including Metema^8,16^. These highland districts are situated on the northeast edges of Lake Tana, with altitudes ranging from 1500 to 2770 m above sea level (masl)^16^. The total population was estimated as 248,807 in Gondar Zuria and 307,967 in East Dembia^26^. Malaria remains a significant public health concern in both districts, with Gondar Zuria experiencing a sharp rise in annual incidence from 24 to 139 cases per 1000 people between 2019 and 2021^15^, while East Dembia reported a 14% prevalence among pregnant women, predominantly caused by *P. falciparum* (73.8%), *P. vivax* (20.7%), and mixed-species infections (5.4%)^27^. These districts experience seasonal malaria transmission, primarily driven by a long rainy season (June–September) and a short season (February–March), with peaking following the rainy seasons, from September to December and from April to May^15,16^.

Metema District, situated within a key development corridor in northwest Ethiopia, has an average altitude of 750 (ranging from 500 to 1000) masl. It is one of nine agricultural investment districts, with a total permanent resident of 154,618. Malaria transmission in Metema occurs year-round, with peaks during harvesting seasons in the spring (September–November), summer (June–August) and winter (December–February)^16,25^. A significant influx of migrant workers during agricultural seasons coincides with peak malaria seasons. Agricultural activities include site clearing, planting, and weeding from May to mid-September, sesame harvesting from late September to December, and sorghum and cotton collection lasting up to 6 months after the initial harvest^8,16,25^. Agricultural worksites typically have limited access to malaria interventions and treatment and employ a high proportion of seasonal migrant workers^4^.

### Study design and sampling

This study used samples and socio-demographic data from a prospective health facility-based case–control study in the highland districts (Dembia and Gonder zuria) and cross-sectional targeted parasite surveys in the lowland district (Metema) (Fig. S1). The case–control study was conducted in 8 health facilities (4 health posts from each district) between October 2022 and April 2023, capturing both peak and minor malaria transmission seasons. The study population included individuals self-presenting to the selected health facilities with malaria symptoms during the study period. Cases included (i) those testing positive for malaria by rapid diagnostic test (RDT) at the time of presentation, and (ii) reclassified controls who later tested positive by quantitative polymerase chain reaction (qPCR). Controls included patients testing negative for malaria by both methods and were frequency matched at the time of recruitment by age category (< 5, 5–14, 15–24, 25–49, ≥ 50 years), gender, health post and month.

The cross-sectional targeted parasite survey in Metema district was conducted during the peak malaria transmission season (October to November 2022). The study population comprised of seasonal workers present at worksites during the study period and resident populations living within a 30-km radius of the farm area. RDTs were performed on-site by healthcare providers to determine malaria infection status according to routine guidelines (using Bioline Malaria Ag P.f/Pan by Abott, STANDARD Q Malaria P.f/P.v Ag by SD Biosensor, or First Response Malaria Ag. P.f./P.v. Card Test). Dried blood spots (DBS) were collected onto Whatman 3MM filter paper for all consenting study participants and dried at ambient conditions.

## Laboratory methods

### Library preparation and sequencing

Genomic DNA was extracted from DBS using the Chelex-Tween 20 method^28^ and quantified using 18S-based qPCR at the Armauer Hansen Research Institute (AHRI) as described by Wampfler et al.^29^. The qPCR targeted the 18S small subunit rRNA gene for *P. falciparum* and *P. vivax* detection and quantification (parasites per µL (p/µL) of blood). Parasite quantification was done using standard curves generated from NF54 ring stage parasites for *P. falciparum* and plasmid constructs for *P. vivax*, with mixed infections quantified by simultaneous detection of both species. Among samples positive for *P. falciparum* or mixed-species infections, 1436 out of 2240 (64.1%), *P. falciparum* parasitemia at > 10 p/µL were selected for sequencing.

Sequencing libraries were prepared from DNA extracts from samples positive for *P. falciparum* (with or without *P. vivax)* by qPCR with the multiplexed amplicon sequencing panel MAD^4^HatTeR^30^ using CleanPlex reagents (Paragon Genomics Inc., Fremont, CA, USA). Primer pool D1.1 was used to amplify 165 high diversity loci in the *P*. *falciparum* genome and targets in the lactate dehydrogenase gene (*ldh*) in *P*. *falciparum*, *P*. *vivax*, *P*. *malariae*, *P*. *ovale* and *P*. *knowlesi*. The multiplex PCR step was conducted for 15 cycles for samples with > 100 parasites/µL and 20 cycles for those with < 100 parasites/µL, and was followed by bead cleaning, digestion and indexing PCR as previously described. In each 96-well plate reaction, four negative controls containing Hypure™ Cell Culture Grade water (Cytiva, Marlborough, MA, USA) and two positive controls were included. Positive controls were prepared by extracting DNA from DBS spotted with cultured W2 strain *P. falciparum* parasites mixed with blood at a 1000 p/µL parasite density. Sample libraries were pooled, quantified and size-checked on a TapeStation 4150 (Agilent Technologies, Santa Clara, CA). Further primer dimer removal was conducted by bead-cleaning or gel-extraction. Libraries were then sequenced with 150 paired-end reads across multiple NovaSeq X runs, with a target coverage of 1000 reads per amplicon per sample.

### Bioinformatic pipeline and quality check of sequences

Raw sequences for all samples were processed using the MAD^4^HatTeR amplicon sequencing pipeline (version 0.2.1, available on Github: https://github.com/EPPIcenter/mad4hatter) and default parameters to infer allelic variants for each locus. Using custom R code (https://github.com/IzzyRou/HRP-manuscript), allele data was quality-checked and filtered for coverage and balancing. Samples were considered successfully sequenced if at least 70% of diversity loci in the panel were represented and at least 50% of those loci had > 100 reads. Samples that failed any of these criteria were reprepared and sequenced. Samples that failed the second sequencing attempt were excluded from analysis. Alleles were filtered by a read threshold of 10, a within host allele frequency (read proportion in a locus) of 1%, and a within population allele frequency (fraction of samples in each population that has the allele) of 1%.

### Data analysis

To generate estimates of within sample parasite diversity, we calculated Complexity of Infection (COI, the estimated number of parasite strains present in an infection) and effective COI (eCOI = 1 + (COI—1) * within-host-relatedness)^31^. Infections were categorized as polyclonal (containing more than one strain) if their eCOI was greater than 1.1. Both were estimated statistically using the *moire* package, which uses a Markov Chain Monte Carlo (MCMC) based approach to jointly estimate complexity of infection and population allele frequencies from polyallelic genomic data^31^. To estimate allele frequencies in the lowland and highland populations, and infer COI using corresponding allele frequencies, the data was first divided into two groups—the case–control highland population and the cross-sectional lowland population—and processed separately before merging the results. Jost’s D and fixation index (FS) were calculated from *moire*-derived allele frequencies as a measure of population differentiation between highland and lowland populations using the *calcFST* within the *polysat* R package. We compared estimates of COI and eCOI between population groups using the Wilcoxon rank sum test and examined their relationship through linear regression of individual estimates. Sequenced parasites from the case control study and parasite survey were then consolidated and reclassified into three population groups for statistical analysis: highland residents, seasonal workers, and lowland residents. For genetic relatedness analysis, parasite genetic data was reclassified according to the human population and recent travel histories, from highlands vs. lowlands to highland residents vs. lowland residents vs. migrant workers. Highlanders in the case–control study were reclassified as seasonal workers if they had engaged in migrant agricultural work or overnight travel for agricultural purposes to Metema within the past 90 days. Similarly, participants from the lowland parasite survey were classified as seasonal workers if they reported overnight agricultural work in Metema outside their kebele of residence within the same timeframe; otherwise, they were classified as lowland residents.

### Clustering analysis

Pairwise genetic relatedness using Identity-by-Descent (IBD) was estimated using the *dcifer* package^32^. *Dcifer* is a statistical tool to estimate relatedness which accounts for multiple strains as a result of co-infection or co-transmission, allowing for more accurate relatedness estimates in populations with polyclonal malaria infections. Relatedness (*r*) values range from 0 to 1. For each sample pair, relatedness estimates were tested against the null hypothesis that unrelated parasites have relatedness (*r*) lower than 0.125, representing three or more out-crossings (*r*_null_ = 0.125, alpha = 0.05). P-values were then corrected for multiple comparisons using the Benjamini-Hochberg (BH) approach with a false discovery rate (FDR) of 0.01^33^. Appropriate relatedness thresholds and adjusted p-value cutoffs were determined empirically (Fig. S2). For all downstream analyses, related sample pairs were subset by a relatedness threshold of 0.25 or higher. The proportion of related sample pairs was calculated for each population, and comparison of proportions were done using Chi-squared tests with Bonferroni (BF) corrections. Clustering of related infections was performed and visualized across the three population groups (Highland resident, Seasonal worker, Lowland resident) using the *igraph* R package^34^, with relatedness estimated by stringent relatedness thresholds of either 0.5 or 1 to focus on highly related pairs that might indicate strong connectivity between populations. Isolates (infections with no related pairs) were excluded from cluster analyses. Since infection pairs that share only common alleles could be mistakenly categorized as identical due to chance (as opposed to identical by descent, i.e. sharing a common ancestor), an additional filter for relatedness estimates with a 95% CI > 0.95–1 was applied for connectivity analyses (~ 59% of identical pairs met this criterion). Degree and centrality were estimated for each infection and summarized over study population, where degree is defined as the number of connections within a cluster, normalized by the number of vertices in the cluster, and closeness estimated by the average shortest distance to another infection within the same cluster^35^.

### Transmission network methods

Transmission network structure was inferred using the *Plasmotrack* package. *Plasmotrack* is a Bayesian approach to inferring directed transmission networks by leveraging genetic and temporal information while allowing for superinfection. The model was fit, constraining each case to have up to 10 possible parent infections with *r* exceeding 0.1, a more permissive relatedness threshold than earlier analyses to identify larger network structures, as estimated by *Dcifer.* Posterior probabilities of parent sets were estimated and used to estimate pairwise probabilities of directed transmission. For the purposes of plotting, the cumulative set of parent sets that exceeded 80% probability were retained and used to calculate pairwise marginal edge probabilities. Pairwise marginal edge probabilities were then filtered to be greater than 50% and plotted (Fig. S6). Transmission connectivity was also summarized by calculating the proportion of edges that originate from intrapopulation groups using the full posterior estimate of pairwise edge probabilities. Inflow proportions were defined as the proportion of edges from each source terminating within a given sink population, and outflow proportions were defined as the proportion of edges from each sink terminating within a given source population (Fig. S7).

## Results

### Study population and amplicon sequencing

A total of 1,436 samples, including 1,154 (80%) *P. falciparum* monoinfections and 282 (20%) *P. falciparum* and *P. vivax* mixed infections by qPCR*,* were sequenced from three population groups. Of those, 1,180 (82%) yielded sufficient reads in ≥ 70% of diversity loci (median number of loci: 165, IQR: 2), including 583 samples from residents of the highland, 534 seasonal workers, and 63 lowland residents. A total of 798 unique microhaplotype alleles were observed, with a median of 2 alleles (min = 1, max = 11) per locus (Table S1). The majority (n = 854) of successfully sequenced samples were *P. falciparum* monoinfections; the remaining were mixed *P. falciparum* infections, which comprised of *P. falciparum infections* mixed with *P. vivax* (n = 280), *P. ovale* (n = 16), *P. malariae* (n = 12), *P. vivax* and *P. ovale* (n = 3), *P. vivax* and *P. malariae* (n = 4), and one sample with coinfection by all species, *P. falciparum*, *P. vivax*, *P. ovale* and *P. malariae* (Table S2).

A small proportion of highland malaria cases (16, 2.7%) reported either agricultural work in the lowlands within the past 3 months or overnight travel to the lowlands for agricultural work within the past month, and these cases were recategorized as seasonal workers. Of participants recruited at lowland worksites, the majority (432, 80.9%) resided outside the Metema district and were reclassified from lowlanders into migrant workers (Table S3).

### Complexity of infection and parasite genetic diversity

**Fig. 2:**
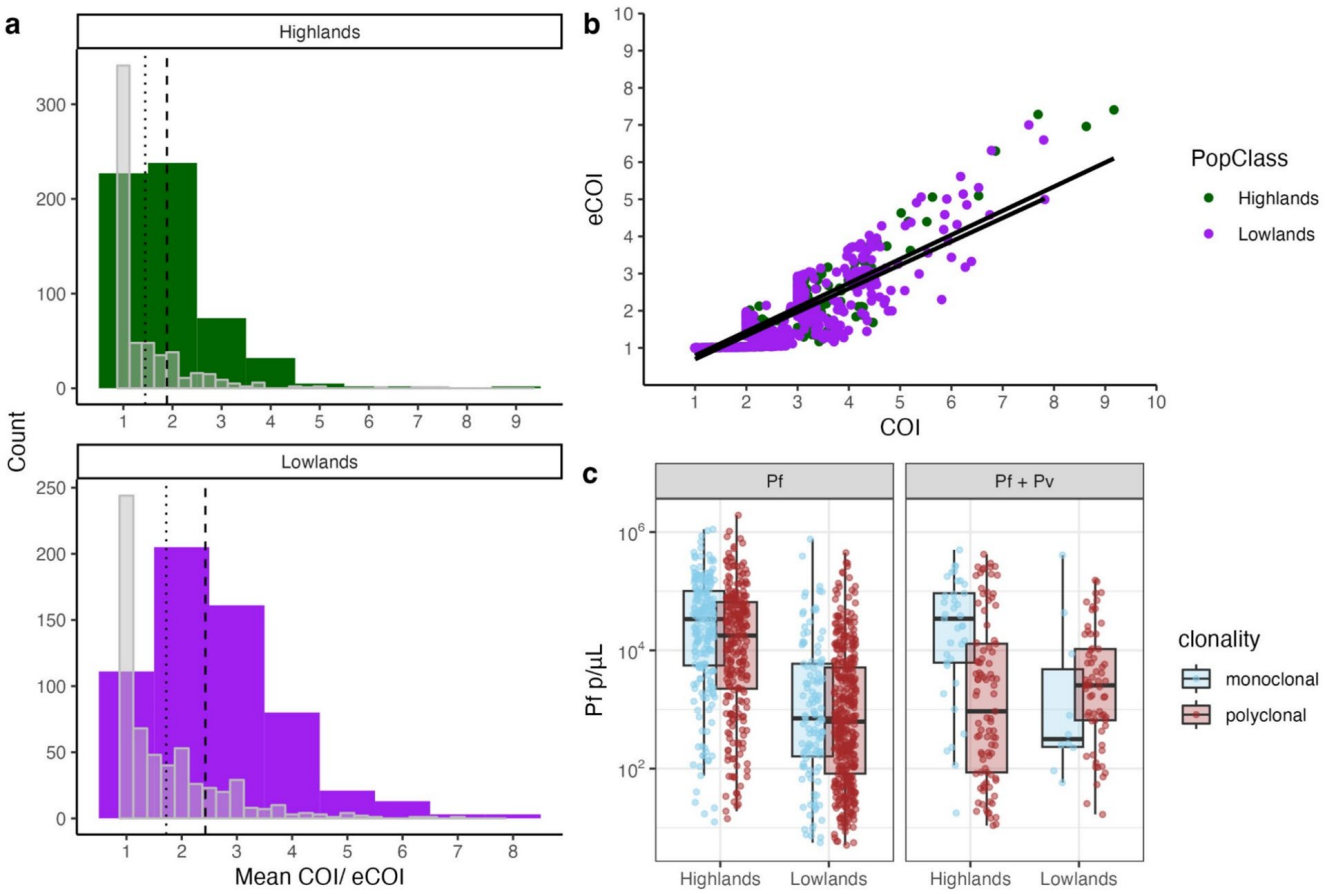
Distribution of COI across different population groups in northwestern Ethiopia. (**a**) COI by population group from highlands (n = 599) and lowlands (n = 581). Thick colored bars represent posterior mean COI at bin width of 1, while overlaid grey bars represent eCOI at bin width of 0.2. Dashed lines represent the median COI, while dotted lines indicate the median eCOI. (**b**) Correlation between COI and eCOI, by population. Linear regression and corresponding equation for each population was determined separately. (**c**) *Plasmodium falciparum* (Pf) parasitemia by mixed and polyclonal status in infections from highlanders and lowlanders.

The highland population had a significantly lower mean (2.00, 95% CI 1.91–2.09) and median (1.88, 95% CI 1.80–1.97) COI compared to the lowland population consisting of both residents and seasonal workers (mean COI = 2.62, 95% CI 2.52–2.72, p < 0.001; median COI = 2.43, 95% CI 2.33–2.52, p < 0.001, Table 1, Fig. 2a). Similarly, the highlands had a lower proportion of polyclonal infections (42%) than in the lowlands (60%, Pearson’s Chi-squared test, p < 0.001, Table 1). Mean eCOI was also significantly lower in the highland (COI = 1.44) compared to the lowland (COI = 1.72) populations (p < 0.001, Table 1, Fig. 2a). The relationship between COI and eCOI (linear regression, slope = 0.627, intercept = 0.134, R^2^ = 0.715), was not different between highlands and lowlands (COI-eCOI:PopClass interaction: slope estimate = -0.015, t = -0.06, p = 0.51) (Fig. 2b).

**Table 1:** Complexity of infection (COI) and effective COI (eCOI) in highland and lowland populations in Northwestern Ethiopia. Mean and median individual COI and eCOI were estimated by *moire*, and polyclonal infections determined if eCOI was greater than 1.1.

Complexity of infection	Highlands N = 599^1^	Lowlands N = 581^1^	P-value^2^
COI mean	2.00 (1.09)	2.62 (1.24)	< 0.001
COI median	1.89 (1.05)	2.43 (1.23)	< 0.001
eCOI mean	1.45 (0.81)	1.72 (0.95)	< 0.001
eCOI median	1.44 (0.81)	1.72 (0.95)	< 0.001
Polyclonal	242 (40%)	360 (62%)	< 0.001

^1^mean (SD); n (%), ^2^Wilcoxon rank sum test; Pearson’s Chi-squared test.

Genetic diversity, assessed using expected heterozygosity (*He*), was similar between the highland (median *He*_*Highland*_ = 0.42 [95% CI 0.39, 0.45]) and lowland (median *He*_*Lowland*_ = 0.46 [95% CI 0.43, 0.49]) populations (Wilcoxon test, p = 0.091). Population differentiation was low, as indicated by FST = 0.014 and Jost’s D = 0.039. A high proportion of mixed species infections in the highlands were monoclonal (77/168, 46%) while infections in the lowlands were highly polyclonal even among mixed species infections (95/148, 64%) (Fig. 2c). The higher proportion of mixed species infections and higher parasite densities in highland infections than in lowland infections within this dataset, however, likely reflects the sampling bias of symptomatic clinical cases in highland population compared to passive sampling within lowland worksites.

### Pairwise genetic relatedness between *P. falciparum* infections

**Fig. 3:**
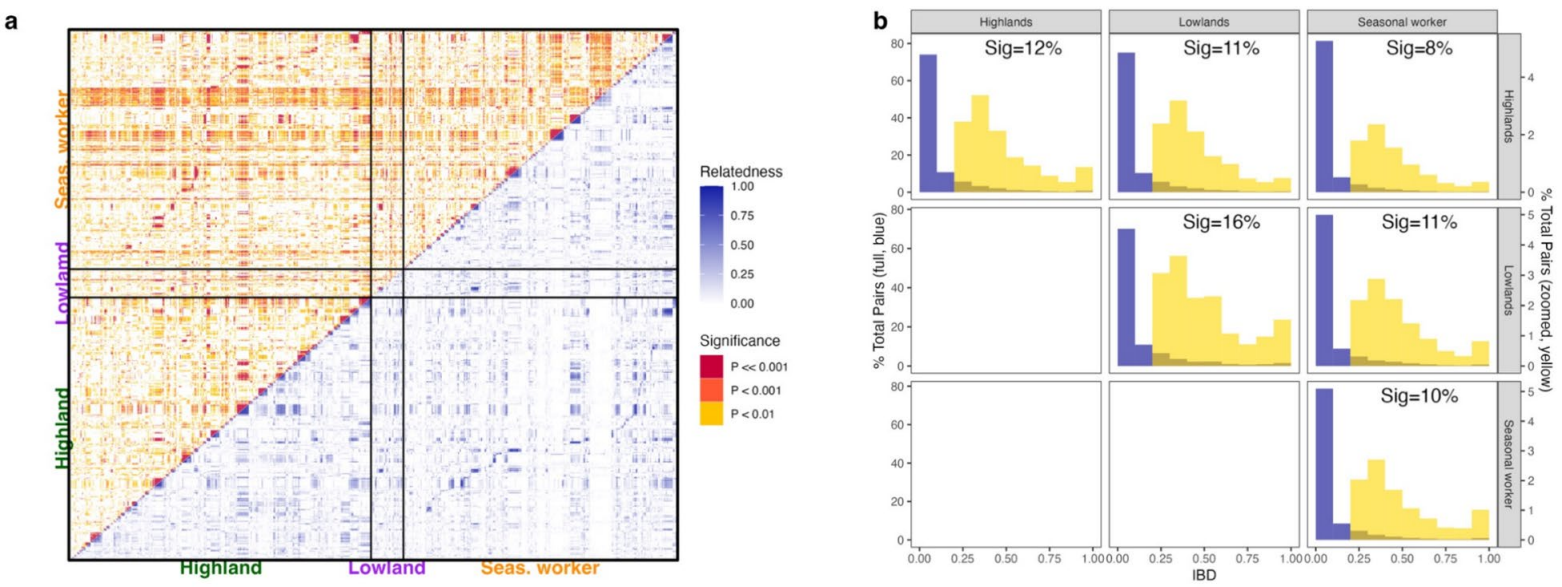
Pairwise relatedness between infections within and between populations. Mean pairwise relatedness by IBD (*r*) between infections from highland residents (n = 583), lowland residents (n = 63) and seasonal workers (n = 534), estimated by *dcifer*. (**a**) Global relatedness clustering between and across populations. (**b**) Percentage of total possible pairs (blue, full distribution) and zoomed percentage of pairs (yellow, *r* ≥ 0.25) at each relatedness interval. “Sig” refers to the percentage of total pairs that were statistically significant with an *r* ≥ 0.25 (represented by the total area of the yellow bars).

High parasite relatedness was observed between infections belonging to the same subpopulation groups (Fig. 3). Across all samples, median and mean between-sample relatedness as measured by IBD was 0.07 and 0.00 for all possible pairs but was 0.65 and 0.69 respectively among statistically significant related pairs. Within highland residents who did not report recent seasonal migrant work to the lowlands, 12% of pairs were related (*r* ≥ 0.25) (Fig. 3a), with a mean *r* of 0.651 among significantly related pairs (p_adj_ < 0.01) (Fig. 3b). Similarly, related pairs constituted 16% and 10% of possible pairs among lowland residents and seasonal workers, with an average *r* of 0.732 and 0.693, respectively. Among related pairs that were statistically significant (p_adj._ < 0.01), identical pairs (*r* = 1) made up a higher proportion of significantly related pairs in the lowland resident (17/104 sig. pairs (16%), 1.0% total pairs) and among seasonal workers (683/4630 (15%) sig. pairs, 0.6% total pairs) compared to infections in the highland population (804/6685 (12%) sig. pairs, 0.5% total pairs; Chi-squared with BF correction, df = 5, p < 0.001) (Fig. 3b).

High parasite relatedness was also observed between infections from individuals in different population groups. There was a slight but significant difference in the proportion of related pairs between lowland residents and seasonal workers (11%, mean *r* = 0.67); highland and lowland residents (11%, mean *r* = 0.631); and highland and seasonal workers (8%, mean *r* = 0.623; Chi-squared with BF correction, p < 0.001) (Fig. 3b). Among related pairings, lowland residents and seasonal workers had a higher proportion of identical pairs (133/911 pairs, 13%) than highland residents and seasonal workers (401/5351 pairs, 6%) or highland and lowland residents (81/974 pairs, 7%). Pairwise relatedness of infections identified within farming worksites varied substantially. Highly related pairs (*r* ≥ 0.8) were observed within specific farms (12 of 33 farms), suggesting localized transmission clusters, while lower median relatedness values (*r* ≤ 0.6) were observed in cross-farm comparisons (Fig. S4), implicating high recombination-driven parasite mixing across farms.

### Parasite connectivity

**Fig. 4:**
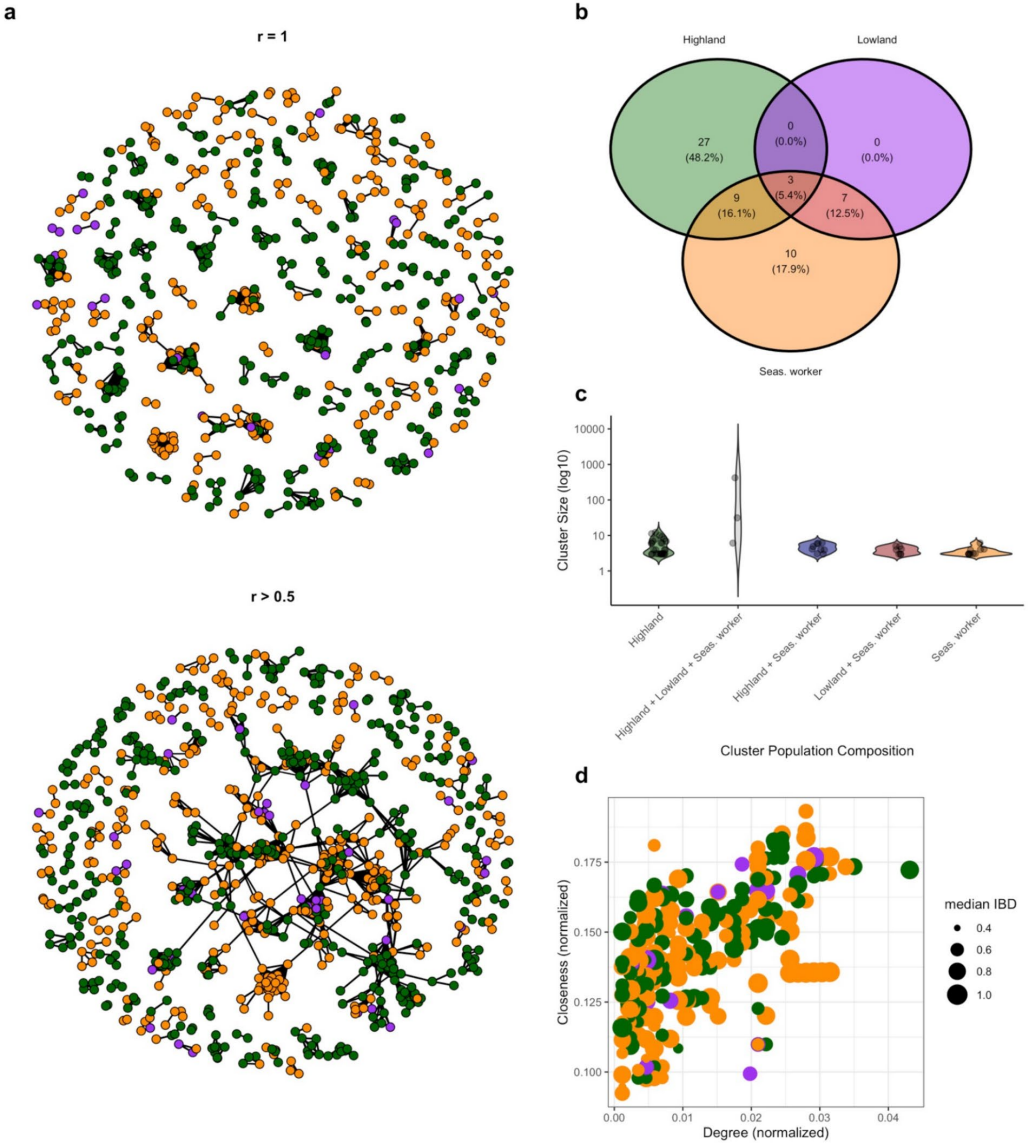
Global clustering of identical and related parasites across study populations. (**a**) Identical (*r* = 1; 95% CI 0.95–1) and related (*r* ≥ 0.5) pairs. Isolates (*r* = 1: n = 560, 47%, (*r* ≥ 0.5: n = 321, 27%) are excluded. (**b**) Clusters were categorized based on the population membership across all members within it. Clusters containing infections from more than one population are represented in overlapping portions of the Venn diagram. (**c**) Cluster membership and cluster size distribution of related (*r* ≥ 0.5) pairs. Cluster size, the number of infections within the cluster, ranged from 3 to 430 infections. (**d**) Centrality of each infection within the 430-member cluster, where size is proportional to the median of all significantly related pairs associated with that infection.

Among the 1180 samples, only 30 had no related pairs at any relatedness level). After limiting the analysis to sample pairs with high confidence (*r* = 1, 95% CI: 0.95–1), a total of 56 clusters (triplet or higher) of genetically identical (*r* = 1) parasites were found, with the majority within highland dwellers (n = 18) and seasonal workers (n = 25); no clusters were found exclusively within lowland residents (n = 0) (Fig. S5). When the r threshold was lowered to r ≥ 0.5), some smaller clusters merged into larger clusters as branches of 1 ≥ r ≥ 0.5 linked them together; clusters encompassing all three populations collapsed from 5 to 3. Half of the related pairs above an *r* threshold of 0.5 (50%), were part of a large 430-member cluster that spanned 36% of all infections across all populations (Fig. 4a). Remaining related clusters (*r* ≥ 0.5) were found primarily within highland cases (n = 27) and within seasonal workers (n = 10), with fewer related clusters including infections from both seasonal workers and lowland residents (n = 7), highland and seasonal workers (n = 9) and inclusive of all populations (n = 3) (Fig. 4b,c). No related clusters were identified that consisted exclusively of samples from lowland residents and highland without seasonal workers. Degree and closeness were similar across all populations (Fig. 4d), suggesting that no one group was more likely to be a central node.

Inferred pairwise transmission probabilities from transmission network estimation also detected substantial clustering across populations, consistent with the findings above (Fig. S6). Estimated inflow proportions suggest that most detected cases are explained by other cases that occur within their own respective population. 71% of edges terminating in highland cases originated from other highland cases, and 80% of edges terminating in seasonal workers originated from other seasonal workers (Fig. S7a). Estimated outflow proportions also suggest differential source-sink dynamics, where 26 and 27% of edges originating from lowland residents and seasonal worker cases respectively terminate in highland cases, while only 3 and 11% of edges originating from highland cases terminate in lowland residents and seasonal worker cases respectively (Fig. S7b).

## Discussion

This study combined high-resolution parasite genomic and epidemiologic data to highlight complex patterns of *P. falciparum* genetic diversity and connectivity within and between Ethiopian highland and lowland populations. These populations are linked by known migration between lowlands and highlands for seasonal agricultural work^8,16^. We observed similar levels of genetic diversity, as measured by heterozygosity, and low population differentiation between highland and lowland populations. This pattern suggests potential gene flow between these populations^36^, likely facilitated by seasonal migration. In addition, the findings point towards high connectivity both within and between highland and lowland populations. Numerous identical parasite clusters were detected within highlanders and a large cluster of highly related samples (*r* ≥ 0.5) linked 36% of infections across populations, indicating frequent human and parasite movement between highland and lowland areas^36,37^. The high prevalence of mixed-species and *P. vivax* infections (39%) observed in this study is consistent with other published estimates^38,39^ and reflects complex malaria ecology transmission dynamics in the region. Findings from this study provide malaria control programs and decision-makers with evidence supporting the role of agricultural corridors in highland transmission dynamics, offering a basis for targeted interventions and surveillance strategies tailored to mobile populations.

This study found that *P. falciparum* infections in the lowland population exhibited a higher mean COI and greater polyclonality compared to those in the highlands. These findings align with the known higher malaria transmission intensities in lowland areas, where endemicity and vector density are significantly elevated compared to highland areas^36,40,41^. The observed polyclonality in lowlands further supports the well-established association between higher transmission intensity and increased multiplicity of infection, as documented across diverse malaria-endemic settings^42–45^. The higher infection complexity observed in this study contrasts with the findings of Holzschuh et al.^36^, who reported that ~ 75% of infections in the highlands were monoclonal, compared to 58% observed here. This discrepancy may be partly attributed to our use of a larger microhaplotype panel (165 vs. 35 loci), which likely improved the sensitivity of detecting multiple parasite strains within infections. Additionally, differences in sampling periods and geographic locations provide context—Holzschuh et al. focused on a single highland district and Ziway in central Ethiopia (2019–2020), while this study used samples from two highland districts and lowlands in 2022–2023. Despite these differences, genetic diversity was comparable both within and between highland and lowland populations.

Addressing human mobility requires a multifaceted approach, encompassing enhanced genetic surveillance, spatial–temporal modeling, migration tracking, and comprehensive vector management^46–48^. In the lowlands, the observed high polyclonality, coupled with high within-host relatedness, likely reflects stable, persistent transmission of related parasite strains^49^. Additionally, localized transmission clusters, especially in agricultural work environments, are supported by studies highlighting specific settings, such as farming areas with temporary breeding habitats formed by seasonal rivers that create numerous riverine pools. These areas foster distinct parasite lineages due to increased human-vector contact during peak malaria transmission seasons^50,51^.

Epidemiological data alone often lacks the resolution to accurately assess parasite connectivity, particularly directionality of transmission (i.e. identifying ‘sources’ and ‘sinks’). This study is consistent with high parasite movement between highland and lowland populations as parasites from both highland and lowland populations showed similar levels of expected heterozygosity, indicating low genetic population differentiation. Such patterns may reflect a combination of factors, including human mobility^52^, shared environmental influences, asymptomatic parasite reservoirs^53^, and vector ecology^16^. A large proportion of highly related parasite pairs were detected both within and between population groups—highlands, lowlands, and seasonal workers—with the latter potentially playing a key role in parasite flow^36^. High relatedness between parasite strains in lowland-migrant and highland-migrant worker pairs further underscores the role of human mobility in shaping parasite population structure, consistent with findings from Tessema et al.^22^ and Bonizzoni et al.^43^. Nonetheless, the observed high relatedness and low population differentiation complicate efforts to determine the directionality of parasite flow (i.e., sources and sinks) or to quantify the precise contributions of seasonal agricultural workers to highland malaria. This is particularly challenging given that parasite siblings were also observed across individuals with no reported travel, implicating alternate means of parasite movement across regions.

We acknowledge several important limitations related to design, laboratory methods, and analysis. First, samples were drawn from two distinct studies with differing designs and sample sizes: highland cases were passively detected, likely symptomatic and higher density, while lowland cases came from cross-sectional surveys. Although there is no evidence of genomic differences between symptomatic and asymptomatic cases, they may differ in treatment-seeking behavior and sequencing success. Second, genotyping faced challenges; higher parasitemia samples were preferentially sequenced, while many low-density samples required resequencing or were excluded entirely. Notably, sample mix-ups (identified by comparing parasite genotypes in DBS re-extractions compared to the originals) in a subset of DBS complicated within-population metadata matching at the individual level; this did not impact overall COI or connectivity results. Third, many identical pairs were called due to the sharing of exclusively common alleles. Moreover, relatedness values of 1 between two polyclonal infections can alternatively be explained by multiple related but not identical parasites if the sum of *r* equals or exceeds 1, which this analysis does differentiate. Fourth, highland data were collected over a longer time period than lowland surveys, and the analysis did not account for temporal bias in sampling. The disparate sampling time frames greatly confound the clustering and transmission network analyses. Future work encompassing more consistent sampling windows and travel histories will allow for finer temporal resolution and determination of directionality across populations.

This study reveals complex patterns of *P. falciparum* genetic diversity and connectivity between Ethiopian highland and lowland populations. High genetic similarity, low population differentiation, and widespread parasite relatedness point to a possible role of human mobility in shaping malaria transmission dynamics. While migration from endemic lowlands likely contributes to highland malaria^37^, with seasonal workers at increased risk of acquiring and reintroducing infections to receptive areas^24^, the precise dynamics of highland transmission require further investigation. In addition to advancing genomic surveillance, this study highlights the need for broader approaches that include *P. vivax* and account for temporal and directional patterns. These findings emphasize the importance of tailored, region-specific malaria control strategies targeting mobile populations and sources of parasite flow across diverse ecological zones. Targeted surveillance of mobile and seasonal populations—through pre-departure screening, targeted chemoprevention, or post-travel follow-up—may help disrupt parasite flow. The genetic homogeneity observed also supports broader, regionally coordinated control efforts over localized interventions. Future research should aim to clarify transmission directionality and the role of seasonal workers in sustaining highland malaria.

## Supplementary Information

Supplementary Information.

## Data Availability

All data are available in the Sequencing Read Archive, accession code PRJNA1252125. All code used for the analysis was written in the R programming language and is available on GitHub (https://github.com/IzzyRou/HRP-manuscript).

## Abbreviations

COI: Complexity of infection
DBS: Dried blood spot
IBD: Identity-by-descent
MAD^4^HatTeR: Multiplex amplicons for drug, diagnostic, diversity, and differentiation haplotypes using targeted resequencing
PCR: Polymerase chain reaction
RDT: Rapid diagnostic test
