# Supplementary material for: High connectivity and low differentiation of Plasmodium falciparum parasite populations in a setting with high seasonal migration
Source: Sci Rep. 2025 Nov 26;15:45386. doi: 10.1038/s41598-025-29271-0 (PMC12749964; doi:10.1038/s41598-025-29271-0)
Supplement: Supplementary file 1 — Supplementary Information. [file 41598_2025_29271_MOESM1_ESM.zip › Supplementary material_FINAL/Supplementary Figures S1-S7.docx]

**High connectivity and low differentiation of *Plasmodium falciparum* parasite populations in a setting with high seasonal migration**

**Supplementary Figures**

**Figure S1**

**
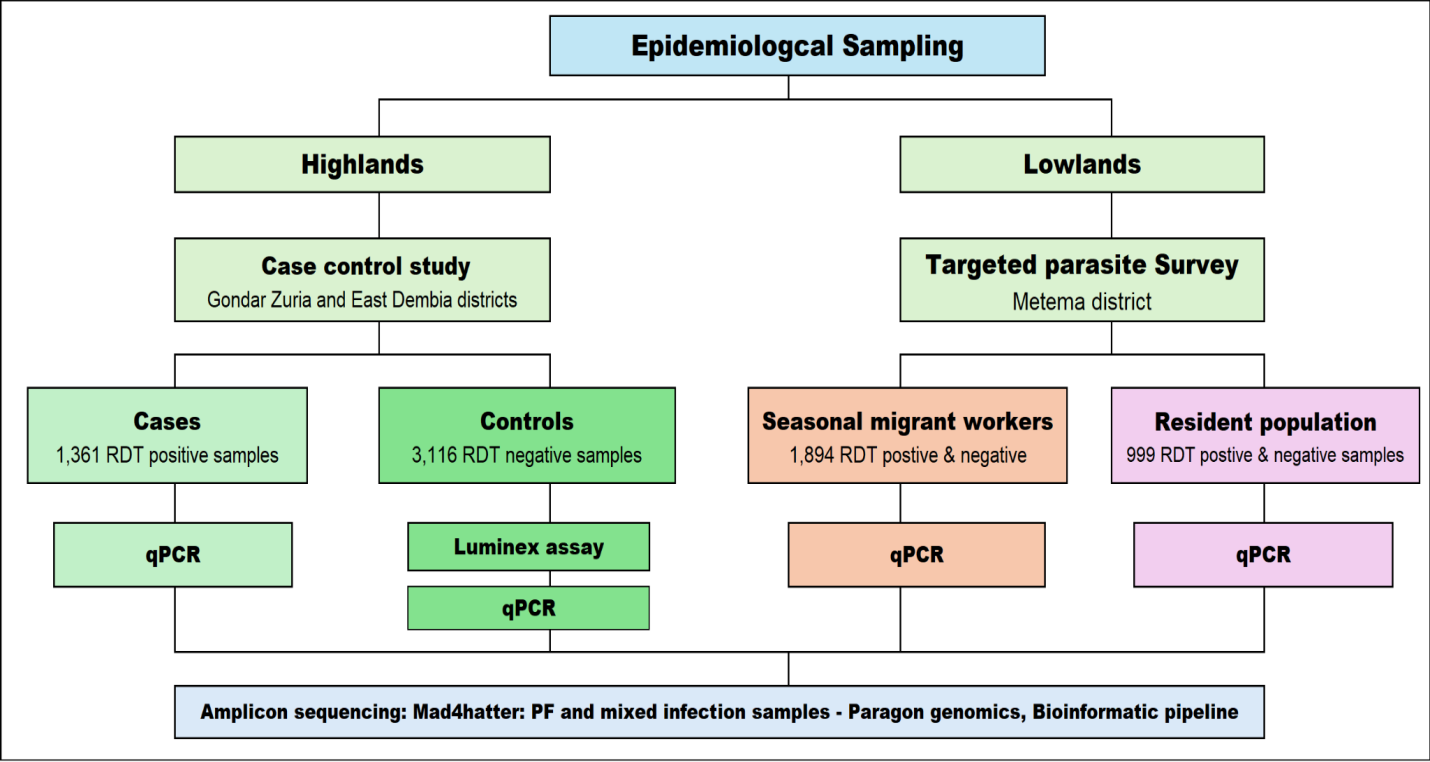
**

**Figure S1:** Schematic diagram depicting the study design and laboratory method used for the highland population, seasonal migrant workers and lowland resident populations, northwestern Ethiopia. MAD^4^HatTeR: Multiplex Amplicons for Drug, Diagnostic, Diversity, and Differentiation Haplotypes using Targeted Resequencing; qPCR: quantitative polymerase chain reaction; RDT: Rapid diagnostic test.


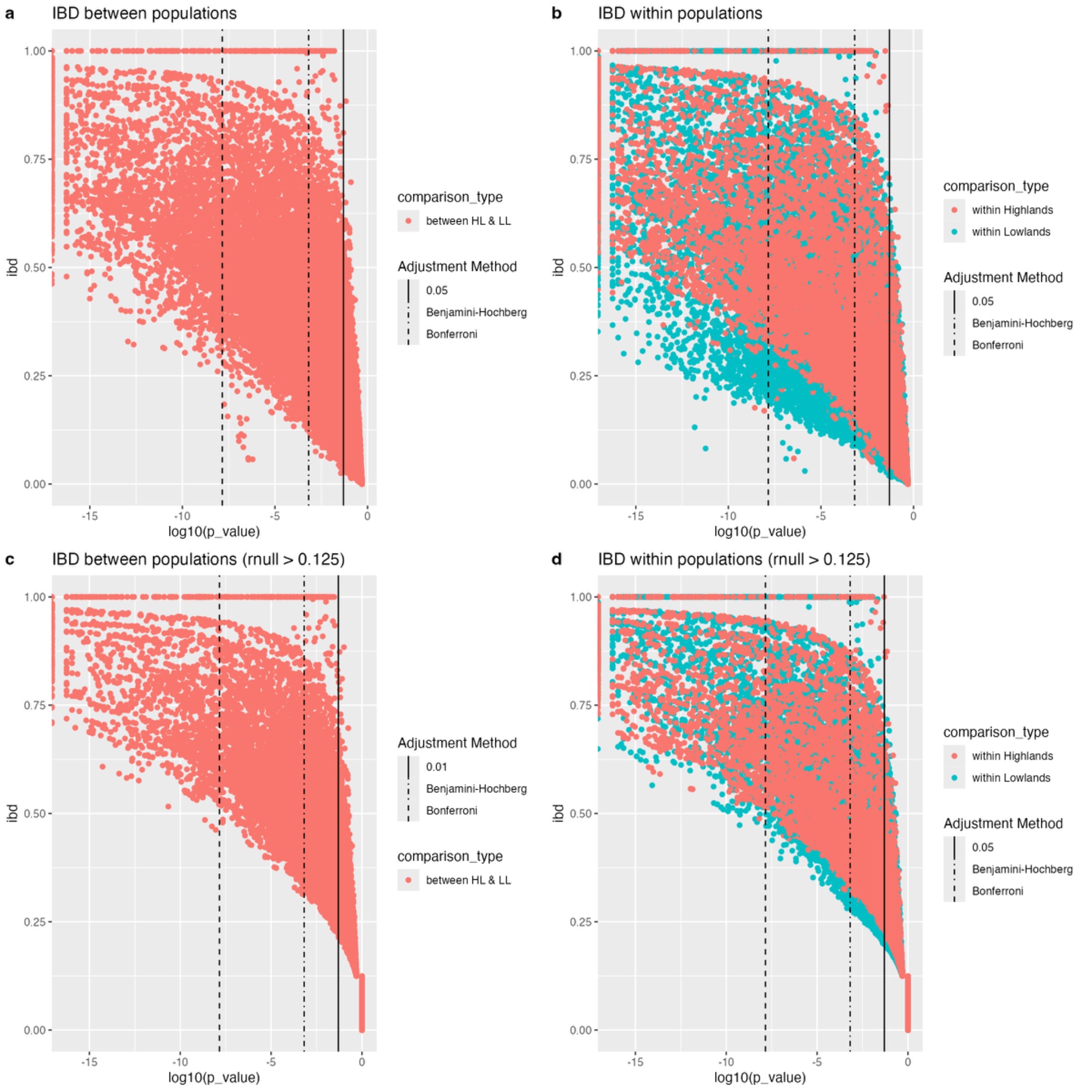


**Figure S2.** P-value adjustments and *r* threshold determination. P-values were adjusted by either Bonferroni or Benjamini-Hochberg methods (FDR = 0.01) to decide which correction to use and at which *r* cut-offs. Vertical lines mark the p-value cut-off and depict how many pairs would be filtered out using the respective corrections. **a)** Relatedness between populations (Highland vs. Lowland), using *r*_null_ = 0. **b)** Relatedness within populations (Highland and Lowland separately), using *r*_null_ = 0. **c)** Relatedness between populations (Highland vs. Lowland), using *r*_null_ = 0.125. **d)** *r* within populations (Highland and Lowland separately), using *r*_null_ = 0.125.


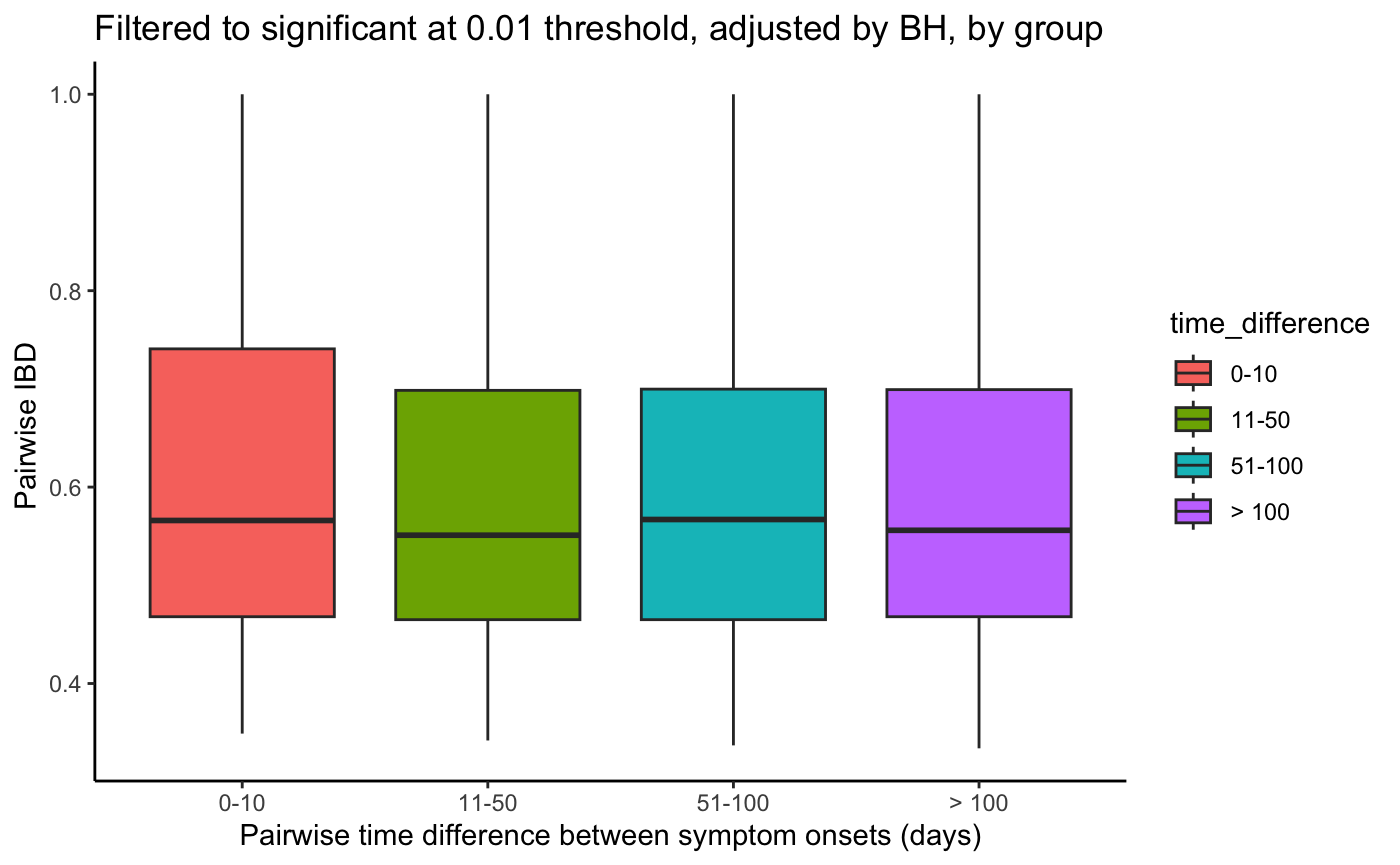


**Figure S3.** Temporal patterns of *r* by date of symptom onset showing the distribution of *r* of sample pairs collected 0–10 days apart, 11–50 days apart, 51–100 days apart or >100 days apart.

**
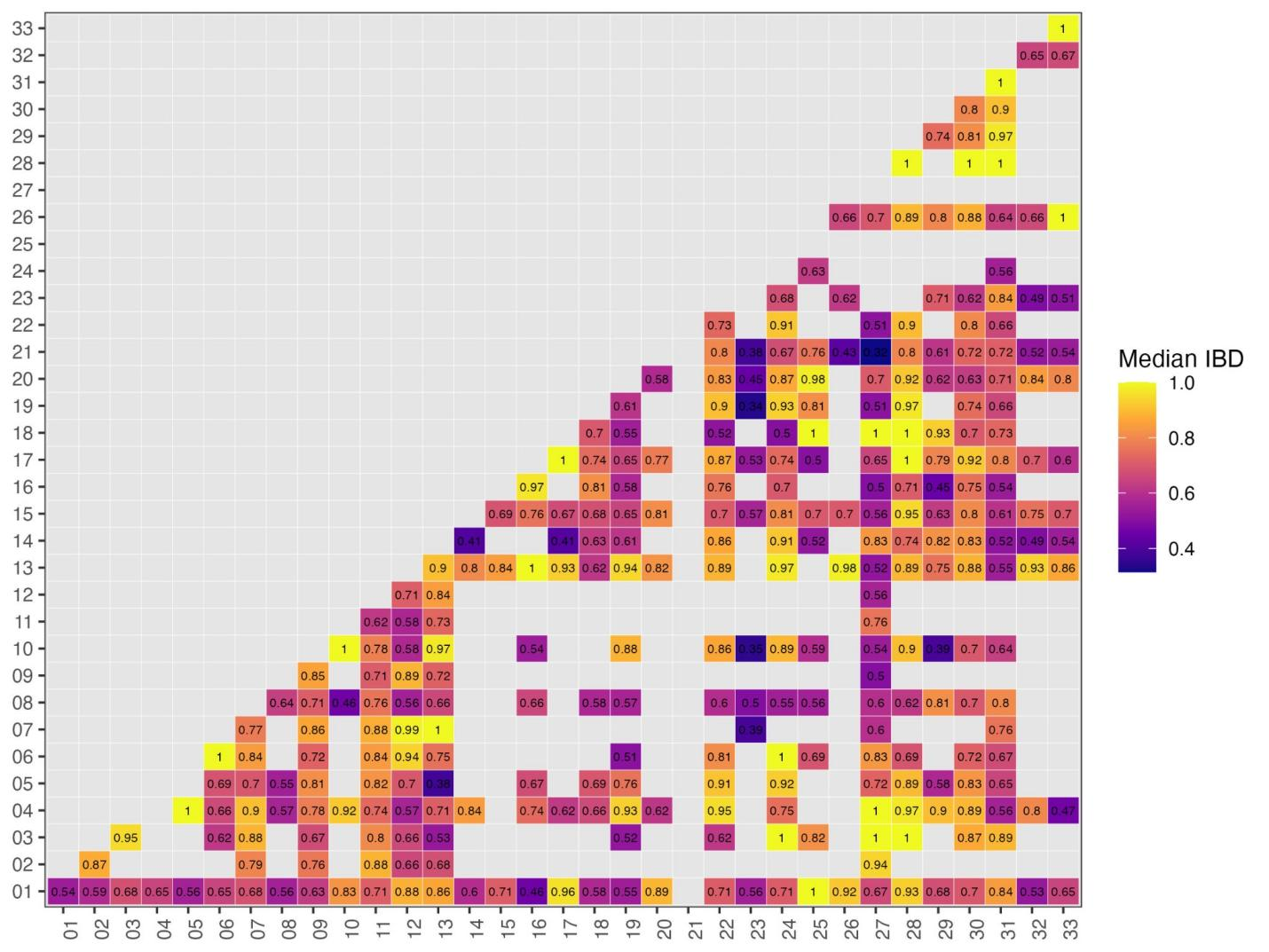
**

**Figure S4.** Median *r* across infections in seasonal workers, by farm. Median *r* values ranged from 0.43 to 1.0, indicating a spectrum of relatedness from moderate to near-identical parasite genotypes within and across different farms. High *r* values (≥ 0.8) were commonly observed within specific farms, suggesting localized transmission clusters or frequent movement of workers among these locations. In contrast, lower median *r* values (≤ 0.6) were observed in cross-farm comparisons, implying reduced parasite sharing or more diverse parasite populations across geographically or epidemiologically distinct farms. Several farm pairings showed particularly strong relatedness. The data points are ordered and asymmetrical, indicating relationships that exist in some farms but not in others. The highest values (*r* = 1.0) were common along the diagonal, indicating that the highest relatedness occurred within, not across farms (**Figure S4**).

**
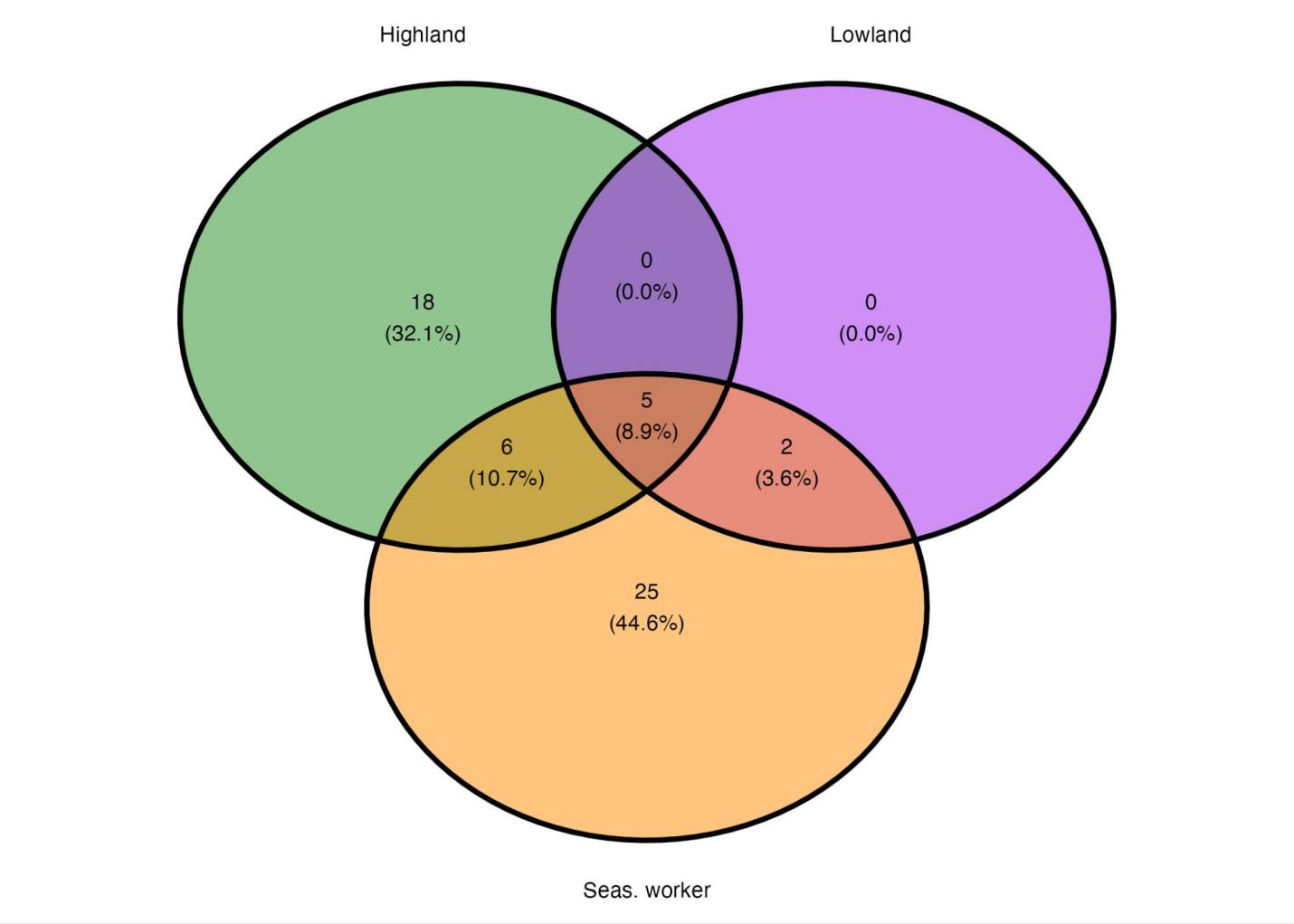
**

**Figure S5.** Cluster membership with *r* threshold of 1.


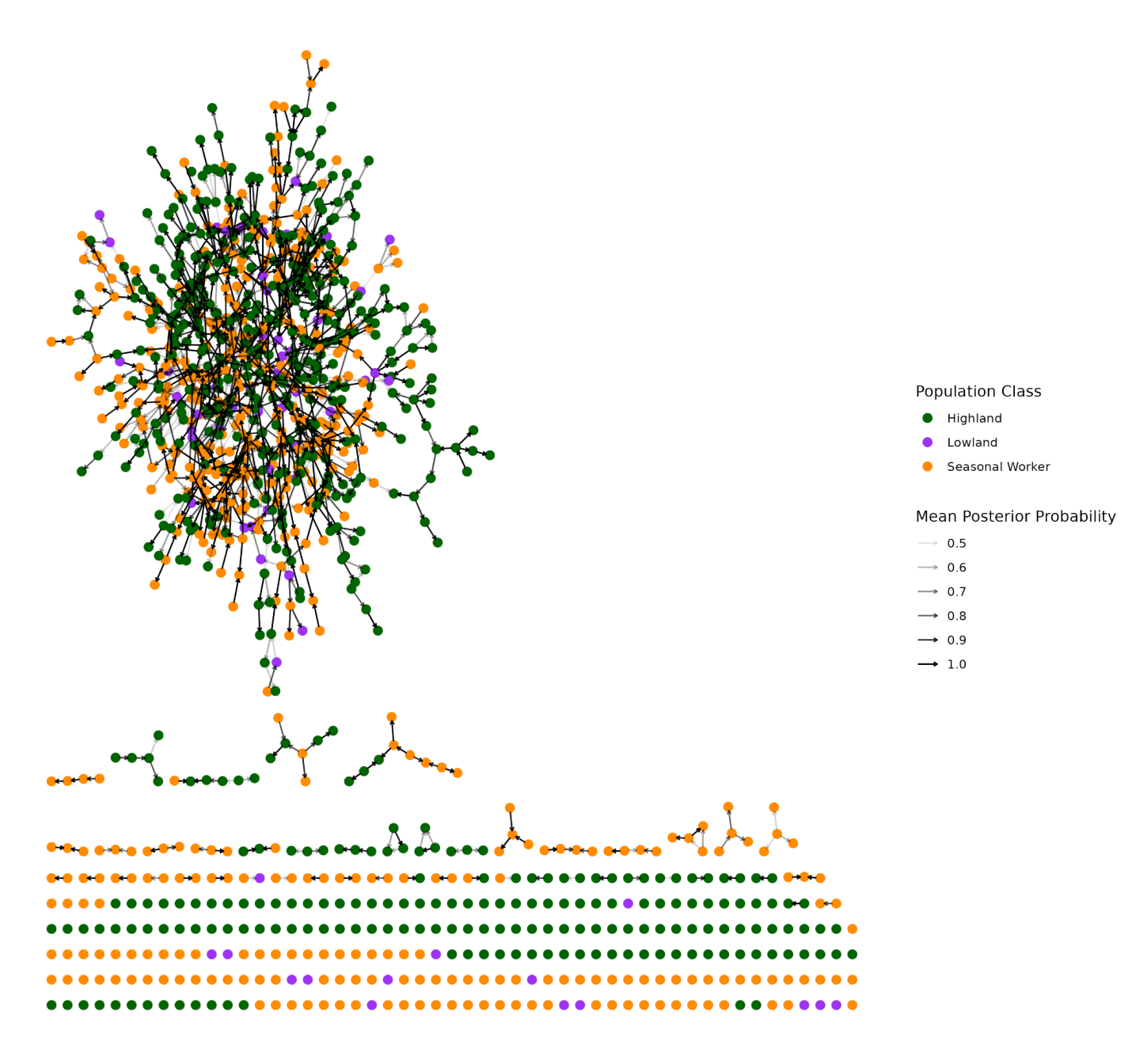


**Figure S6.** Estimated transmission network.


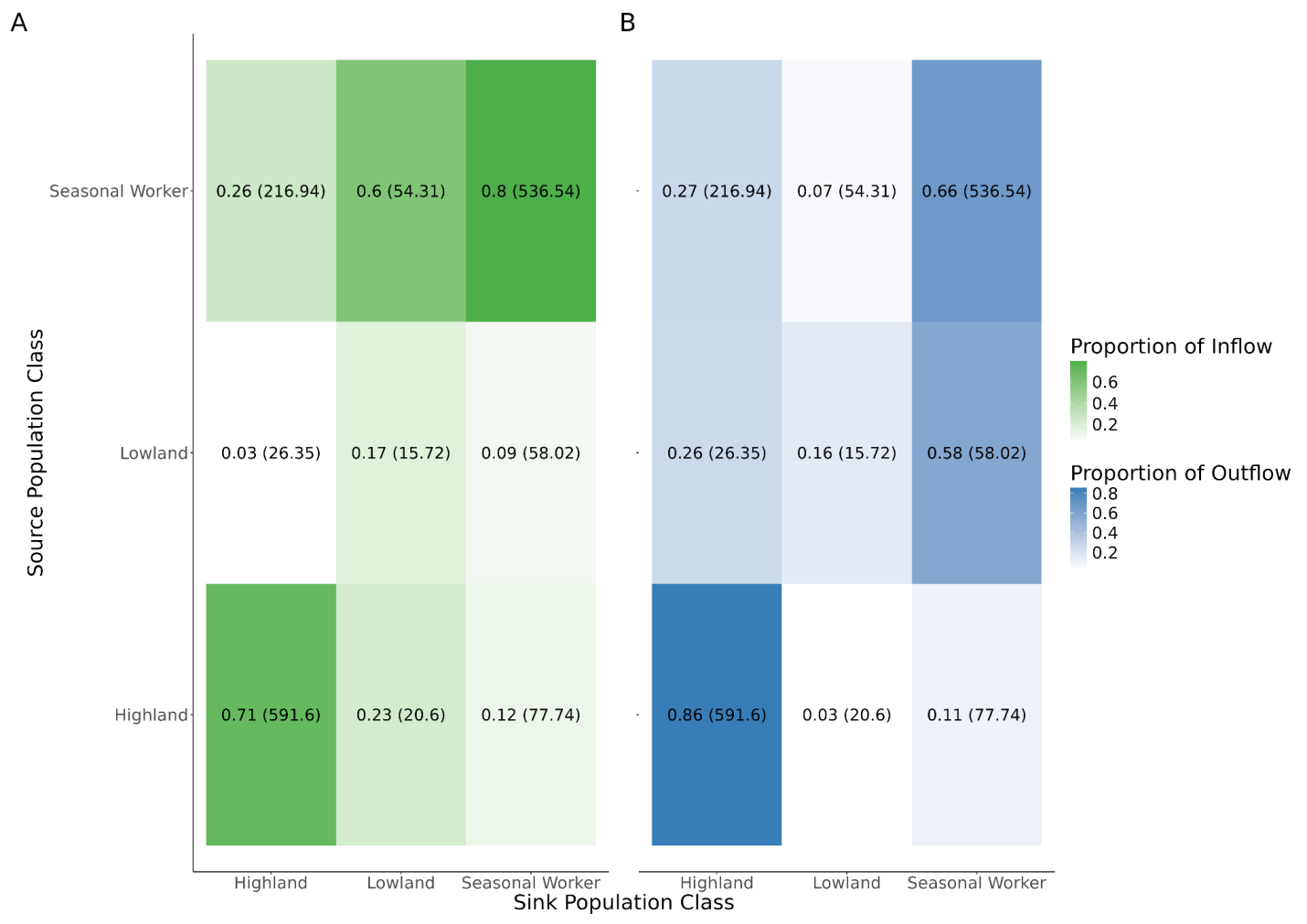


**Figure S7.** Estimated transmission flow between populations. Proportions are calculated with respect to the total incoming (A) or outgoing (B) edges of each population. The total number of edges between populations are indicated in parentheses.
